# Correction: Shibly et al. Analysis of Cerebral Small Vessel Changes in AD Model Mice. *Biomedicines* 2023, *11*, 50

**DOI:** 10.3390/biomedicines12010104

**Published:** 2024-01-04

**Authors:** Abu Zaffar Shibly, Abdullah Md. Sheikh, Makoto Michikawa, Shatera Tabassum, Abul Kalam Azad, Xiaojing Zhou, Yuchi Zhang, Shozo Yano, Atsushi Nagai

**Affiliations:** 1 Department of Neurology, Faculty of Medicine, Shimane University, 89-1 Enya-cho, Izumo 693-8501, Japan; shibly@med.shimane-u.ac.jp (A.Z.S.); akzad88@mib.jnu.ac.bd (A.K.A.); zhoug93@med.shimane-u.ac.jp (X.Z.); zhangyuchi1014@icloud.com (Y.Z.); 2 Department of Biotechnology and Genetic Engineering, Mawlana Bhashani Science and Technology University, Santosh, Tangail 1902, Bangladesh; 3 Department of Laboratory Medicine, Faculty of Medicine, Shimane University, 89-1 Enya-cho, Izumo 693-8501, Japan; abdullah@med.shimane-u.ac.jp (A.M.S.); tabassum@med.shimane-u.ac.jp (S.T.); syano@med.shimane-u.ac.jp (S.Y.); 4 Department of Biochemistry, Graduate School of Medical Sciences, Nagoya City University, Nagoya 467-8601, Japan; michi@med.nagoya-cu.ac.jp

## Error in Figure

In the original publication [1], an error was identified in Figure 5b. More precisely, the photomicrographs of the 6-month WT (third picture in the upper row) and the 3-month J20 (second picture in the lower row) in Figure 5b were found to be identical. To correct this issue, the erroneous photomicrograph has been replaced with the correct representation of the 3-month J20 (second picture in the lower row). The corrected Figure 5b is provided below.

The authors state that the scientific conclusions are unaffected. This correction was approved by the Academic Editor. The original publication has also been updated.

## Figures and Tables

**Figure 5:**
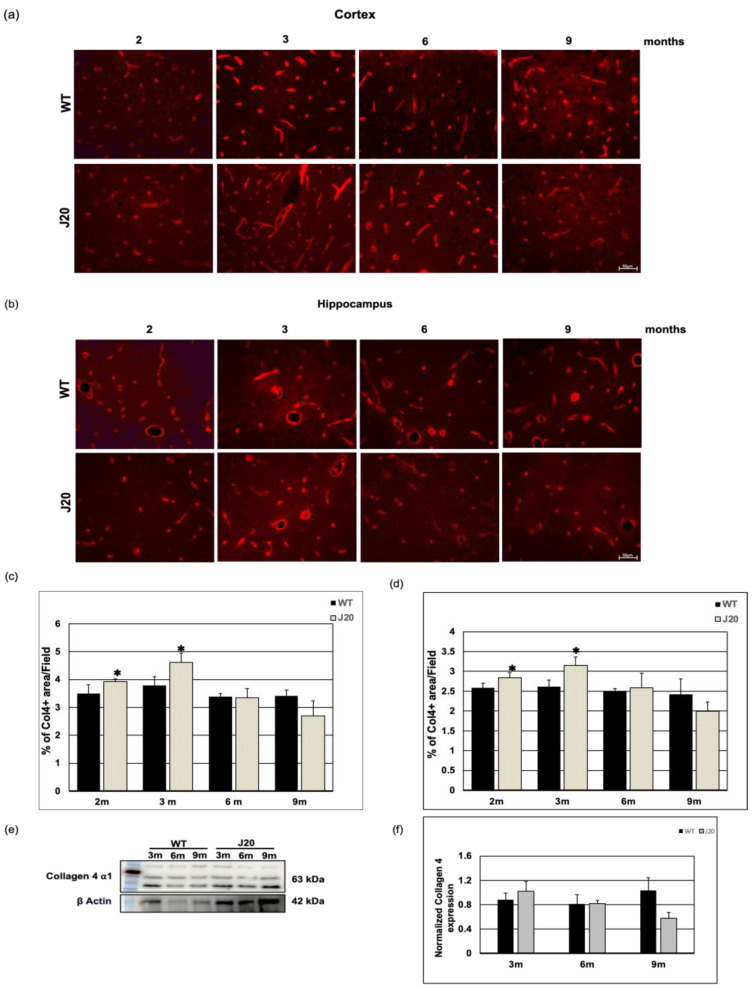
Time-dependent changes in collagen 4 levels in J20 mice brain. (**a**,**b**) Immunoreactivity of collagen 4 in the cortex (**a**) and hippocampus (**b**) of WT and J20 mice at 2, 3, 6, and 9 months of age are shown. Collagen 4 immunoreactivity was found around the vessel in WT and J20 mice brains. (**c**,**d**) Densitometric analysis was carried out using ImageJ, and the quantified data are expressed as a percent of collagen 4-positive areas per field (×40) in the cortex (**c**) and hippocampus (**d**). (**e**,**f**) A representative immunoblot of collagen 4 protein in the hippocampus of WT and J20 mice brains at 3, 6, and 9 months of age is shown here in (**e**). Quantified data of β actin normalized collagen 4 in the hippocampus at 3, 6, and 9 months of age are shown in (**f**). Numerical data are presented here as average ± SD, and statistical significance is denoted as * *p* < 0.05 vs. age-matched WT. Col4 = Collagen 4. m indicates month. Scale bar = 50 µm.
