# Supplementary material for: Analysis of Cerebral Small Vessel Changes in AD Model Mice
Source: Biomedicines. 2022 Dec 25;11(1):50. doi: 10.3390/biomedicines11010050 (PMC9855388; doi:10.3390/biomedicines11010050)
Supplement: Supplementary file 1 [file biomedicines-11-00050-s001.zip › biomedicines-2034455-supplementary.pdf]

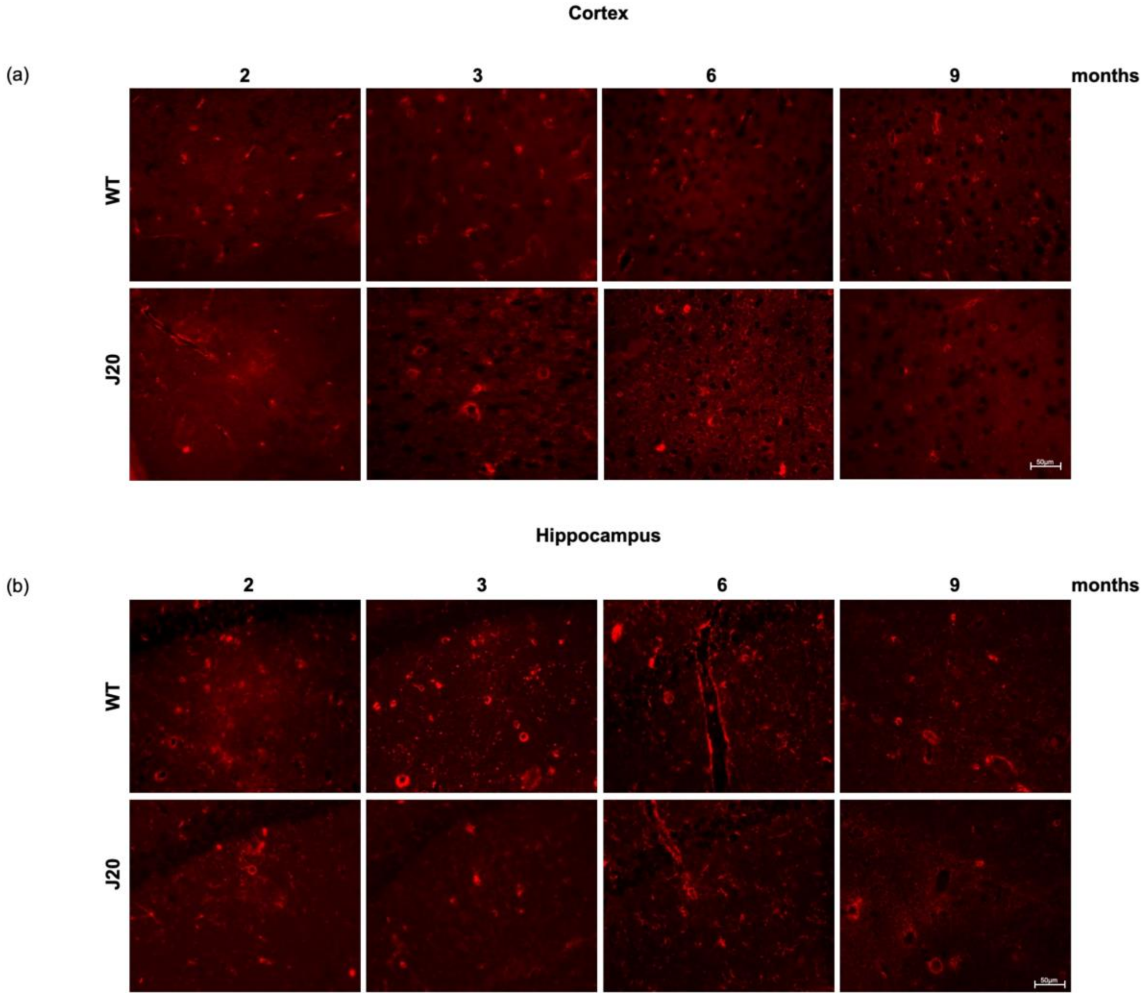

Figure S1. Claudin-5 immunoreactivity in the cortex (a) and hippocampus (b) of both WT and J20 mice brain at 2, 3, 6, and 9 months of age.

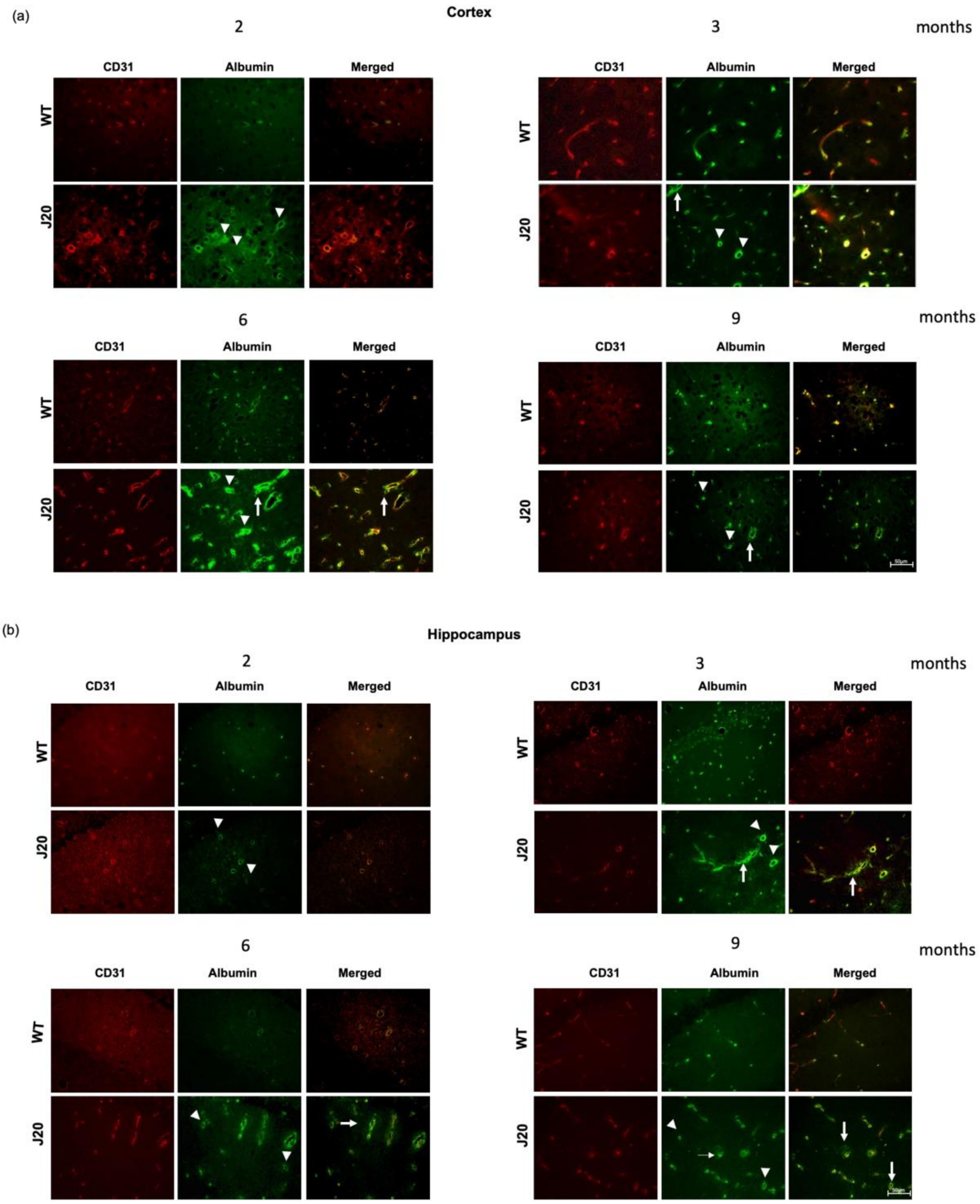

Figure S2. Double fluorescence staining for the markers of the vessel, endothelial cell CD31 (red), and albumin (Green) in the cortex (a) and Hippocampus (b) of both WT and J20 mice at 2, 3, 6, and 9 months of age. Albumin was positive for vessel, and it was intravascular in WT mice at every time point from 2 month to nine months of age. In J20 mice brain, albumin was observed to be leaked out of vessel (white arrows) or perivascular region (white arrowhead). Scale bar, = 50 μm.

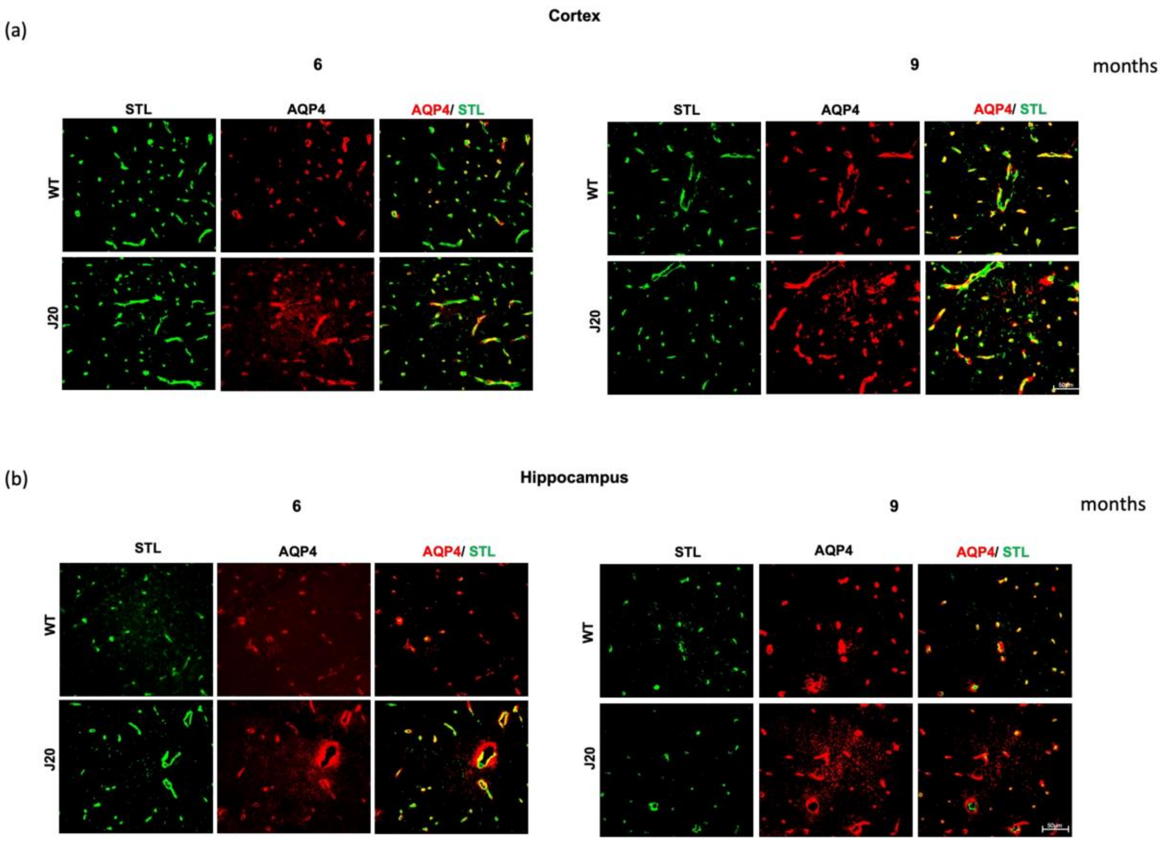

Figure S3**.** AQP4 immunoreactivity for polarization in the cortex (a) and hippocampus (b) of both WT and J20 mice brains at 6 and 9 months of age. Scale bar = 50μm.

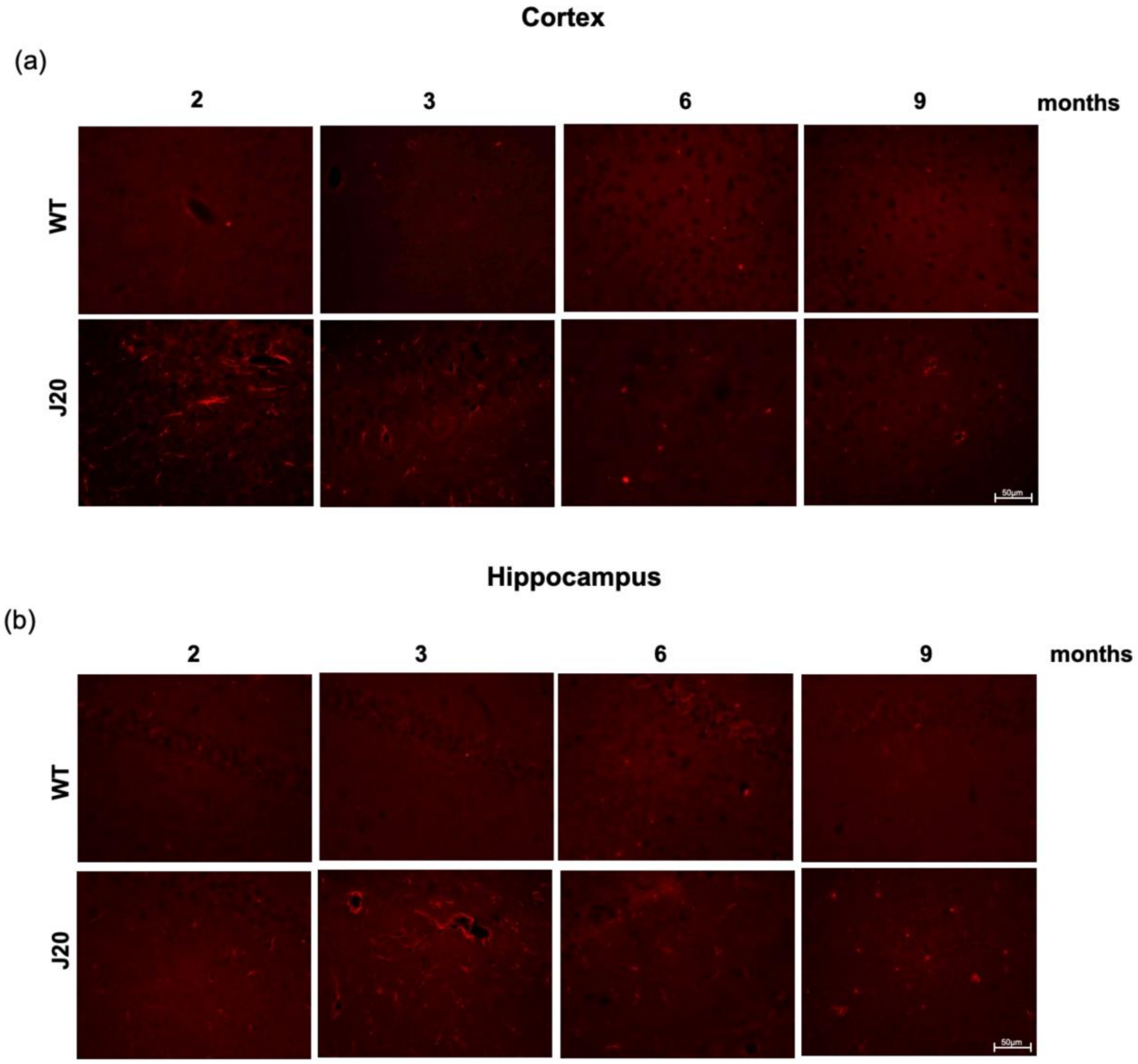

Figure S4. Immunofluorescence immunoreactivity of VEGF in the cortex (a) and hippocampus (b) of both WT and J20 at 2, 3, 6, and 9 months of age. Scale bar = 50μm.
